# Risperidone Administration Attenuates Renal Ischemia and Reperfusion Injury following Cardiac Arrest by Antiinflammatory Effects in Rats

**DOI:** 10.3390/vetsci10030184

**Published:** 2023-02-28

**Authors:** Yang Hee Kim, Tae-Kyeong Lee, Jae-Chul Lee, Dae Won Kim, Hyun-Jin Tae, Joon Ha Park, Ji Hyeon Ahn, Choong-Hyun Lee, Moo-Ho Won, Seongkweon Hong

**Affiliations:** 1 Department of Surgery, Kangwon National University Hospital, School of Medicine, Kangwon National University, Chuncheon 24289, Republic of Korea; 2 Department of Food Science and Nutrition, Hallym University, Chuncheon 24252, Republic of Korea; 3 Department of Neurobiology, School of Medicine, Kangwon National University, Chuncheon 24341, Republic of Korea; 4 Department of Biochemistry and Molecular Biology, and Research Institute of Oral Sciences, College of Dentistry, Kangnung-Wonju National University, Gangneung 25457, Republic of Korea; 5 College of Veterinary Medicine and Biosafety Research Institute, Iksan 54596, Republic of Korea; 6 Department of Veterinary Medicine and Institute of Animal Transplantation, Jeonbuk National University, Iksan 54596, Republic of Korea; 7 Department of Anatomy, College of Korean Medicine, Dongguk University, Gyeongju 38066, Republic of Korea; 8 Department of Physical Therapy, College of Health Science, Youngsan University, Yangsan 50510, Republic of Korea; 9 Department of Pharmacy, College of Pharmacy, Dankook University, Cheonan 31116, Republic of Korea

**Keywords:** antipsychotic drug, blood urea nitrogen, creatinine, histopathology, inflammatory cytokines, lactate dehydrogenase

## Abstract

**Simple Summary:**

Risperidone has been reported to show other beneficial effects instead of its original effectiveness. This experiment was conducted for the effects of risperidone on renal ischemia and reperfusion injury (IRI) following cardiac arrest. The increased levels of serum blood urea nitrogen, creatinine, and lactate dehydrogenase after cardiac arrest were significantly decreased by risperidone treatment. IRI-induced histopathological injury was attenuated by risperidone administration, showing that pro-inflammatory and anti-inflammatory cytokine immunoreactivities were apparently controlled by risperidone administration. Based on these findings, risperidone administration after cardiac arrest can protect kidneys from IRI via anti-inflammatory effects.

**Abstract:**

Multi-organ dysfunction following cardiac arrest is associated with poor outcome as well as high mortality. The kidney, one of major organs in the body, is susceptible to ischemia and reperfusion; however, there are few studies on renal ischemia and reperfusion injury (IRI) following the return of spontaneous circulation (ROSC) after cardiac arrest. Risperidone, an atypical antipsychotic drug, has been discovered to have some beneficial effects beyond its original effectiveness. Therefore, the aim of the present study was to investigate possible therapeutic effects of risperidone on renal IRI following cardiac arrest. Rats were subjected to cardiac arrest induced by asphyxia for five minutes followed by ROSC. When serum biochemical analyses were examined, the levels of serum blood urea nitrogen, creatinine, and lactate dehydrogenase were dramatically increased after cardiac arrest, but they were significantly reduced by risperidone administration. Histopathology was examined using hematoxylin and eosin staining. Histopathological injury induced by cardiac arrest was apparently attenuated by risperidone administration. Furthermore, alterations in pro-inflammatory cytokines (interleukin-6 and tumor necrosis factor-α) and anti-inflammatory cytokines (interleukin-4 and interleukin-13) were examined by immunohistochemistry. Pro-inflammatory and anti-inflammatory cytokine immunoreactivities were gradually and markedly increased and decreased, respectively, in the kidneys following cardiac arrest; however, risperidone administration after cardiac arrest significantly attenuated the increased pro-inflammatory cytokine immunoreactivities and the decreased anti-inflammatory cytokine immunoreactivities. Collectively, our current results revealed that, in rats, risperidone administration after cardiac arrest protected kidneys from IRI induced by cardiac arrest and ROSC through anti-inflammatory effects.

## 1. Introduction

It is known that renal diseases in domestic animals are commonly caused by aging, congenital factors, pathogenic infections and toxicosis. In the present study, renal injury was induced by whole-body ischemia and reperfusion in rats, which is pathogenetically different from the renal diseases commonly diagnosed in domestic animals. In this regard, this experiment was conducted from the aspects of comparative medicine.

Cardiac arrest (CA) refers to the sudden loss of heart function that results in an abrupt halt of effective blood flow to the body. The morbidity and mortality due to CA have increased worldwide [1]. Many studies have focused on myocardial dysfunction and brain injury following CA and return of spontaneous circulation (ROSC) [2,3,4,5]. Dysfunctions in various organs are common after ROSC following cardiopulmonary resuscitation (CPR) from CA [6]. Nevertheless, few studies on renal ischemia and reperfusion injury (IRI) following CA and ROSC (CA/ROSC) have been performed. The recovery of blood flow after CA/ROSC can cause renal injury, known as renal IRI [7,8]. Renal IRI following CA/ROSC can cause acute kidney dysfunction, further contributing to high mortality [9]. It has been reported that kidney injury in patients with CA/ROSC occurs frequently, occurring in ~50% of cases of CA/ROSC [10].

The pathophysiology following renal IRI is complex and not fully understood yet; however, renal inflammation is accepted as one of the important pathogenic components [11,12]. Inflammatory cascade in an ischemic kidney is induced a few hours after IRI and lasts for a long time (days or weeks) as a delayed reaction to the damage [13,14]. In the early phase after renal IRI, macrophages promote inflammation and amplify IRI through the release of pro-inflammatory cytokines (tumor necrosis factor-α, TNFα and interleukin-4, IL4) [15,16]. However, in the late phase after renal IRI, macrophages express anti-inflammatory cytokines (IL4 and IL13) and play an important role for the repair process [17,18].

Risperidone (Risp, a benzoxazole derivative), as a second-generation antipsychotic drug, has been primarily used to treat schizophrenia and bipolar disorder [19,20]. It has been reported that Risp induces hypothermia in patients with mental disorders, such as schizophrenia [21,22]. In recent experimental studies with Risp, the administration of Risp after brain-IRI-induced hypothermia and effectively protected neurons from IRI in gerbil hippocampus and rat spinal cord [23,24].

It is generally admitted that body temperature influences, to a certain extent, the outcomes of IRI in patients after CA/ROSC [25,26,27]. It has been reported that therapeutic hypothermia can improve the survival rate and the neurological outcomes of post-CA patients for several years [28,29]. Hypothermia can attenuate ischemia-induced tissue damage in important organs, including the heart, kidney, and liver [30,31]. It has been reported that Risp induces hypothermia in patients with mental disorders, such as schizophrenia [21,22]. However, the effects of Risp on the survival rate and kidney IRI in a rat model of CA/ROSC has not been studied. Therefore, this study investigated the effects of Risp on mortality and renal histopathology using rats with CA/ROSC. In addition, with regard to the mechanisms of the effects, changes in inflammatory cytokines were evaluated in rat ischemic kidney treated with Risp.

## 2. Materials and Methods

### 2.1. Experimental Protocol and Animals

The protocol of this experiment (approval number, KW-200113-1) was authorized by the Institutional Animal Care and Use Committee affiliated with Kangwon National University (Chuncheon, Republic of Korea) on 18 February 2020. All animal procedures were conducted in compliance with the guideline of the “Current International Laws and Policies” which is a part of the “Guide for the Care and Use of Laboratory Animals” [32]. Male rats (Sprague-Dawley; 10-week-old; body weight, 310 ± 7 g) were supplied from the Experimental Animal Center, an affiliated institution of Kangwon National University (Chuncheon, Republic of Korea). Until the rats acclimatize to the laboratory environment, they were kept in a conventional room for two weeks controlled at about 24 °C of room temperature and 55% of relative humidity was used and provided steady dark and light cycle every 12 h and freely accessible pellet feed and water.

Four groups were used for this study: (1) Sham-vehicle group (*n* = 7) received sham operation and was administered a vehicle (saline), (2) Sham-Risp group (*n* = 7) received sham operation and was administered Risp, (3) CA-vehicle group (*n* = 21) received CA/ROSC operation and was administered vehicle, and (4) CA-Risp group (*n* = 21) received CA/ROSC operation and was administered Risp.

### 2.2. Operation of CA/ROSC and Risp Administration

**Figure 1:**
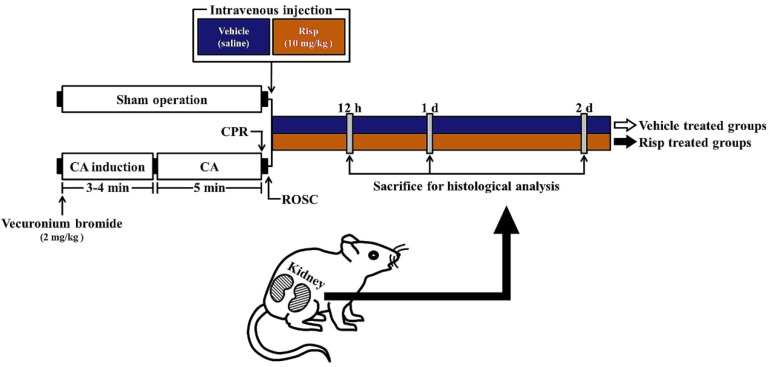
Experimental design and timeline. In this experiment, all rats underwent sham or CA/ROSC operation followed by administration of saline (vehicle) or Risp (10 mg/kg). The rats were profoundly anesthetized and sacrificed at 12 h, one day, and two days after CA/ROSC. Thereafter, the obtained kidneys were used for the analyses. CA, cardiac arrest; CPR, cardiopulmonary resuscitation; ROSC, return of spontaneous circulation; Risp, risperidone.

As shown in Figure 1, the CA/ROSC operation was conducted according to previous studies [24,33] with modifications. Briefly, the rats were fasted for eight hours excepting free access to water and anesthetized with 2.5–3% isoflurane (Hana Pharmaceutical Co., Ltd.; Seoul, Republic of Korea). Under the anesthesia, each rat was placed on a surgical board in the supine position; the trachea of the rat was intubated with a 14-gauge cannula through a tracheotomy under mechanical ventilation using a ventilator (Harvard Apparatus, Holliston, MA, USA).

A PE-50 catheter (ADInstruments Ltd., Sydney, Australia) was canulated in the left femoral artery to measure mean arterial pressure (MAP) and to take a blood sample. The MAP was monitored with MLT 1050/D (ADInstruments Ltd.). Another PE-50 catheter was canulated in the right femoral vein for the delivery of fluids and drugs. The catheters were intermittently cleaned with sterilized normal saline. A pulse oximetry saturation probe (Nonin Medical Inc., Plymouth, MN, USA) was connected to the left foot in order to monitor peripheral oxygen saturation, as saturation of percutaneous oxygen (SpO_2_). For the monitoring of electrocardiogram (ECG), three electrode leads (GE healthcare, Chicago, IL, USA) were, respectively, connected to both forelimbs and left hind limb. To control body temperature, the rats were monitored with a TR-100 rectal temperature probe obtained from Fine Science Tools (Foster City, CA, USA) and controlled at 37 ± 0.5 °C (normothermia) using a thermometric blanket (Harvard Apparatus). After collection of baseline data and stabilization for five minutes, a single dose of 2 mg/kg vecuronium bromide (Reyon Pharmaceutical Co., Ltd., Seoul, Republic of Korea) was intravenously injected to induce respiratory paralysis and immobilize the rats. Three to four minutes later, the ventilator was removed and the endotracheal tube was clamped to induce asphyxia. Asphyxial CA was defined by confirming pulseless electric activity of ECG and less than 25 mmHg of MAP. ROSC was performed immediately after confirming CA as follows. CPR by chest compression and ventilation was begun five minutes of the CA for 60 s according to manual chest compression. Simultaneously, 0.005 mg/kg of epinephrine (Dai Han Pharmaceutical Co., Ltd., Seoul, Republic of Korea) and 1 mEq/kg of sodium bicarbonate (Daewon Pharmaceutical Co., Ltd., Seoul, Republic of Korea) were intravenously injected. In this experiment, the rats with sham operation received the same operation except CA/ROSC.

For the experimental groups, 10 mg of Risp (Sigma-Aldrich, St. Louis, MO, USA) was dissolved in 0.85% saline, and vehicle (saline) or Risp was intravenously injected after CA/CPR operation.

The rats with CA/ROSC were respired spontaneously one hour after ROSC and the hemodynamics became stable. After confirming the stability, the cannulated catheters, endotracheal intubation, electrode leads and oximetry probe were removed. Finally, the rats were subcutaneously administered isotonic saline (20 mL/kg/d) containing 5% dextrose until the rats were able to drink and eat by themselves.

### 2.3. Biochemical Analysis of Serum

An intraperitoneal injection of pentobarbital sodium (180 mg/kg; JW Pharmaceutical Co., Ltd., Seoul, Republic of Korea) was used to anesthetize all animals. Blood sampling was conducted from the abdominal veins of all rats. Serum was collected by blood centrifugation (2774× *g*, 15 min, 4 °C) and preserved at −80 °C until the analysis. According to a method outlined by the International Federation of Clinical Chemistry [34], the levels of blood urea nitrogen (BUN), creatinine, and lactate dehydrogenase (LDH) were determined using an automated Olympus AU2700 Analyzer (Olympus, Optical Co., Tokyo, Japan). This analysis was conducted in triplicate using fresh serum.

### 2.4. Hematoxylin and Eosin (HE) Staining

HE staining and analysis were performed according a previous study [35] with minor modification. In short, at the designated points in time after CA, all rats were deeply anesthetized via intraperitoneal injection of 180 mg/kg of pentobarbital sodium (JW Pharmaceutical Co.) and flushed with heparinized saline by transcardial perfusion until all blood was fully cleared. Continuously, the rats were flushed with 4% paraformaldehyde (in 0.1 M phosphate buffer (PB), pH 7.4). The kidneys were removed and postfixed in the 4% paraformaldehyde for three days. The kidneys were trimmed, dehydrated, cleared and embedded into paraffin wax. Paraffin sections (six-μm thickness) were made using Leica microtome (Wezlar, Germany). Thereafter, the sections were deparaffinized, reacted with hematoxylin (ThermoScientific, Waltham, MA, USA), washed, and reacted with Eosin Y (ThermoScientific). Finally, the sections were dehydrated, cleared and coverslipped with Canada balsam (Kanto Chemical Co., Inc., Tokyo, Japan).

For examination of histopathological changes in the kidneys, the stained tissue slides were observed and captured the images using BX53 light microscope (Olympus, Tokyo, Japan). The scoring was performed in accordance with injured level as zero to five scale: zero, none; one, 0–10%; two, 11–25%; three, 26–45%; four, 46–75%; and five, 76–100%.

### 2.5. Immunohistochemistry

To compare alterations in pro-inflammatory (TNFα and IL6) and anti-inflammatory (IL4 and IL13) cytokines between the four groups, TNFα, IL4, IL6 and IL13 expressions were evaluated by a standard immunostaining method. In brief, as described in the “Section 2.4”, the paraffin sections were deparaffinated and hydrated. Then the sections were incubated in a blocking reagent containing 1% hydrogen peroxide in methanol and 5% horse and/or rabbit serum (in 100 mM PBS, pH 7.4) for 25 min at room temperature, respectively. The sections were then immunoreacted with each primary antibody (Table 1) for 12 hours at 4 °C. Afterward, the sections were reacted with secondary antibody for one hour at room temperature and incubated in avidin-biotin complex (diluted, 1:250; Vector Laboratories, Burlingame, CA, USA). Thereafter, the sections were washed with 0.1 M PBS (pH 7.4) and visualized using 0.05% 3,3′-diaminobenzidine tetrahydrochloride (Sigma-Aldrich Co., St. Louis, MO, USA) in 100 mM PBS containing 0.1% hydrogen peroxide. Then the sections were dehydrated, cleared and covered with cover glasses and Canada balsam (Kanto Chemical Co.).

The TNFα, IL4, IL6 and IL13 immunostained structures were compared between the four groups as follows. Five sections per rat were selected, and each image was taken like the method described in the “Section 2.4”. The image was converted into eight-bit grey scale (from zero to 255) image and assessed for grey scale intensity. The immunoreactive intensity was calculated using Image J 1.46 obtained from National Institutes of Health (Bethesda, Maryland, MD, USA) and presented as ROD as percentage (ROD of Sham + vehicle group, 100%).

### 2.6. Data and Statistical Analyses

In this study, all experiments were carried out in a randomized manner. Using G*Power 3 software [36], sample size was calibrated with an alpha error of 0.05 and a power of >80%, resulting in two animals per group for the minimum. The results obtained from this experiment were expressed as means ± standard error of the mean (SEM). To calculate identical SEM, Bartlett test was performed, and to evaluate normal distribution, a Kolmogorov and Smirnov test was carried out. All data were analyzed using SPSS 18.0 (SPSS, Chicago, IL, USA). Kaplan–Meier method and the log-rank test were used to analyze cumulative survival rate. One- and two-way ANOVA was used to analyze MAP and SpO2. A post hoc Tukey’s test was used to analyze the significance of statistical differences among all groups. *p* values less than 0.05 were statistically considered significant.

## 3. Results

### 3.1. Body Temperature and Survival Rate

**Figure 2:**
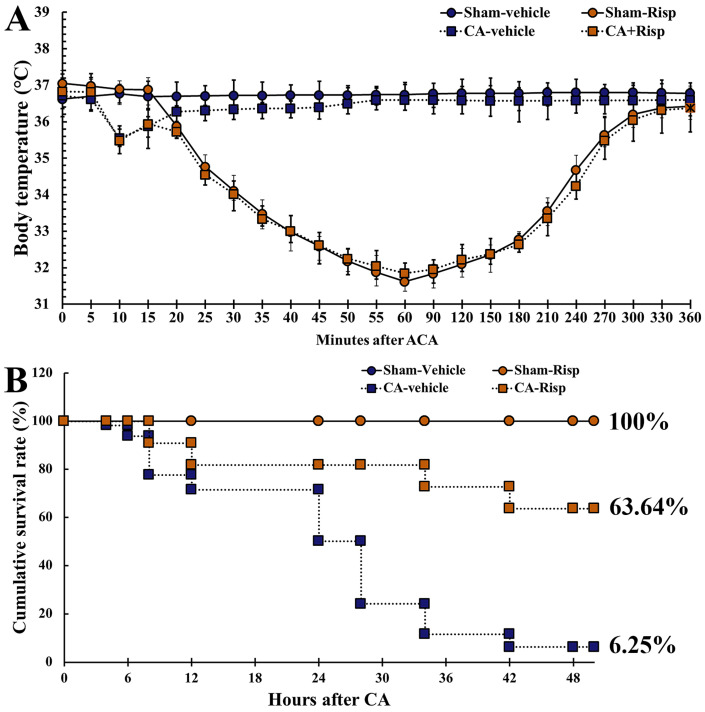
(**A**,**B**) Changes in body temperature (**A**) measured during and after CA/ROSC for six hours in four groups. Cumulative survival rate (**B**) assessed via using Kaplan–Meier analysis in four groups after CA/ROSC. The bars indicate the means ± SEM.

In this experiment, body temperature was recorded before and after CA. Body temperature before CA was similar to the baseline obtained in the Sham + vehicle group. In the Sham-Risp and CA-Risp groups, body temperature was significantly different from that shown in the Sham-vehicle group. In the two groups, a significantly low body temperature (33 ± 0.5 °C) was observed from one to two hours after CA (Figure 2A). Thereafter, body temperature was spontaneously and gradually increased to 37 ± 0.5 °C with intermittently shivering (Figure 2A).

In each group, the survival rate of the rats was determined two days after ROSC by Kaplan–Meier analysis which demonstrated a significant difference in the survival rate. In the Sham-vehicle group, the survival rate was 100%. The survival rate in the CA-vehicle group was 6.25%; however, the rate in the CA-Risp group was 63.64% (Figure 2B).

### 3.2. Levels of BUN, Creatinine and LDH

**Figure 3:**
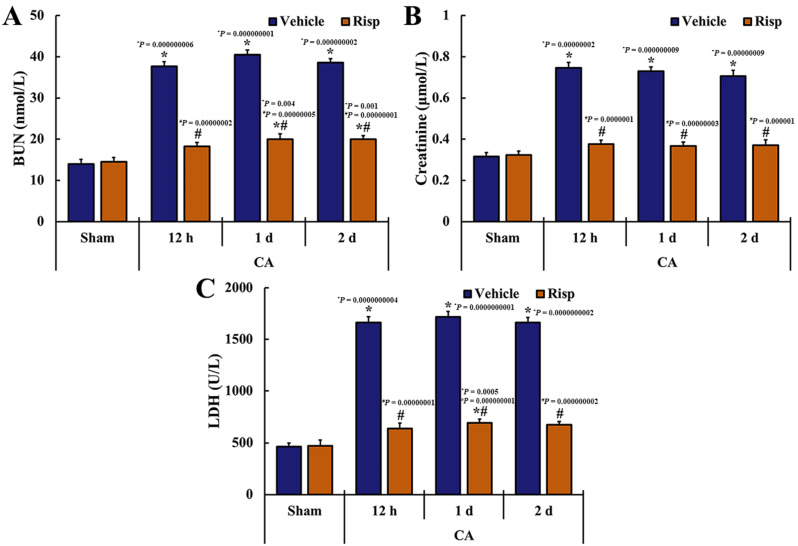
Changes in levels of BUN (**A**), creatinine (**B**), and LDH (**C**). The levels were determined at 12 h, one day, and two days after CA/ROSC. The levels of BUN, creatinine, and LDH in the CA-Risp group were significantly (*p* < 0.05) low when compared with the CA-vehicle group. The bars indicate the means ± SEM (*n* = 7, respectively; * *p* < 0.05 vs. Sham-vehicle group; # *p* < 0.05 vs. CA-vehicle group).

The levels of serum BUN, creatinine, and LDH were analyzed to evaluate the renal function of the experimental rats (Figure 3). The rats of the CA-vehicle group showed significant increases (*p* < 0.05) in BUN (SEM: 1.17, 1.16, 1.12, 1.03 at sham, 12 h, 1 d and 2 d after CA/ROSC), creatinine (SEM: 0.020, 0.027, 0.022, 0.028 at sham, 12 h, 1 d and 2 d after CA/ROSC), and LDH (SEM: 35.9, 55.2, 53.1, 52.3 at sham, 12 h, 1 d and 2 d after CA/ROSC) levels when compared with those obtained from the Sham-vehicle group. In the CA-Risp group, however, the serum BUN (SEM: 1.03, 0.96, 1.30, 0.88 at sham, 12 h, 1 d and 2 d after CA/ROSC), creatinine (SEM: 0.019, 0.020, 0.020, 0.025 at sham, 12 h, 1 d and 2 d after CA/ROSC), and LDH (SEM: 57.4, 49.6, 34.7, 33.8 at sham, 12 h, 1 d and 2 d after CA/ROSC) levels were significantly (*p* < 0.05) decreased from 12 h to two days after CA/ROSC when compared with the CA-vehicle group.

### 3.3. Histopathology by HE Staining

**Figure 4:**
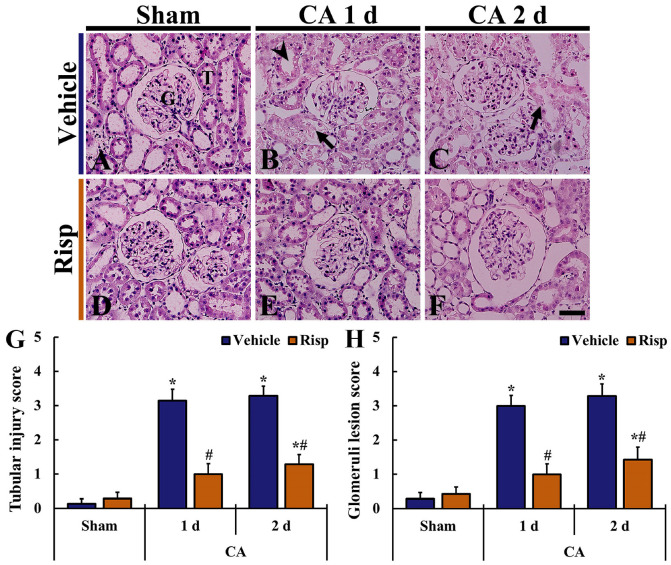
HE staining in the kidney of the Sham-vehicle (**A**), CA-vehicle (**B**,**C**), CA-Risp (**D**), and CA-Risp (**E**,**F**) groups one day and two days after CA/ROSC. In the rats of CA-vehicle group, the patchy denudation of renal tubular cells with loss of brush border (arrows), vacuoles in tubular cells (arrowheads), and tubular dilation are shown, which are more severe at 48 h after CA/ROSC. In the rats of the CA-Risp group, however, the CA/ROSC-induced alterations are apparently attenuated. G, glomerulus; T, tubule. Scale bar = 100 µm. (**G**,**H**) Tubular injury score (**G**) and glomeruli lesion score (**H**). The bars indicate the means ± SEM (*n* = 7, respectively; * *p* < 0.05 vs. Sham-vehicle group; ^#^ *p* < 0.05 vs. CA-vehicle group).

Based on the HE staining, in the Sham-vehicle group, renal tissue had typical structures, with the infiltration of only a few inflammatory cells (Figure 4A). In the CA-vehicle group, one days after CA, renal lesion (patchy denudation of renal tubular cells with loss of brush border, vacuoles in tubular cells, dilated lumen of tubules, glomerular capillary dilatation, inflammatory cell infiltration, etc.) was apparent when compared with those in the Sham-vehicle group (Figure 4B). Two days after CA/ROSC. renal injury was significantly increased (Figure 4C,G,H).

The morphology of renal tissue in the Sham-Risp group was similar to that of the Sham-vehicle group (Figure 4D). In the CA-Risp group, kidney injury was apparently attenuated one and two days after CA/ROSC (Figure 4E,F). In particular, Risp treatment after CA/ROSC significantly attenuated the damage of brush border and the expansion of the tubules, decreased vacuolization in the tubular cells, and improved glomerular injury as compared with those obtained from the CA-vehicle group (Figure 4E–H).

### 3.4. Pro-Inflammatory Cytokine Immunoreactivity

#### 3.4.1. IL6 Immunoreactivity

**Figure 5:**
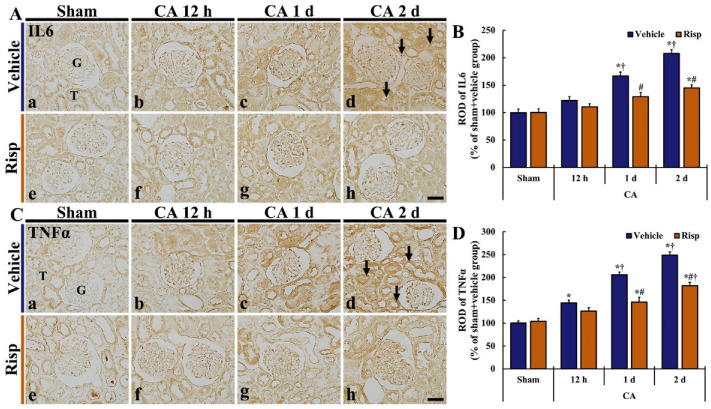
(**A**,**C**) Immunohistochemical staining of IL6 (**A**) and TNFα (**C**) in the kidney of the Sham-vehicle (**a**), CA-vehicle (**b**–**d**), Sham-Risp (**e**), and CA-Risp (**f**–**h**) groups at 12 h, one day, and two days after CA/CPR. IL6 and TNFα immunoreactivities in the CA-vehicle group are gradually and significantly enhanced after CA/CPR: On day 2 after CA/ROSC, strong IL6 and TNFα immunoreactivities are found in tubular cells (arrows). However, in the CA-Risp group, IL6 and TNFα immunoreactivities are significantly lower than those shown in the CA-vehicle group. G, glomerulus; T, tubule. Scale bar = 100 µm. (**B**,**D**) RODs of IL6 (**B**) and TNFα (**D**) immunoreactivities. The bars indicate the means ± SEM (*n* = 7, respectively; * *p* < 0.05 vs. Sham-vehicle group; ^#^ *p* < 0.05 vs. CA-vehicle group; ^†^ *p* < 0.05 vs. Pre-time point of corresponding group).

Weak IL6 immunoreactivity was observed in the renal tissue of the Sham-vehicle group (Figure 5Aa). IL6 immunoreactivity in the CA-vehicle group was slightly increased (122.0% of Sham-vehicle group) 12 h after CA/ROSC, showing that the increased immunoreactivity was shown mainly in tubular cells (Figure 5Ab,B). Thereafter, IL6 immunoreactivity was more increased (166.8% of Sham-vehicle group) on day 1 after CA/ROSC and highest (207.9% of Sham-vehicle group) on day 2 after CA/ROSC, showing that strong IL6 immunoreactivity was shown in tubular cells on day 2 after CA/ROSC (Figure 5A(c,d),B).

In the Sham-Risp group, IL6 immunoreactivity in the renal tissue was similar to that shown in the Sham-vehicle group (Figure 5Ae). In the CA-Risp group, IL6 immunoreactivity was also gradually increased with time after CA/ROSC, but the immunoreactivity was significantly lower (77.4% and 69.7% of CA-vehicle group on day 1 and 2, respectively, after CA/ROSC) than that in the CA-vehicle group (Figure 5B).

#### 3.4.2. TNFα Immunoreactivity

Weak TNFα immunoreactivity was detected in the kidney of the Sham-vehicle group: the immunoreactivity was generally expressed in tubular cells (Figure 5Ca). In the rats of the CA-vehicle group, TNFα immunoreactivity was slightly increased in the tubules at 12 h (Figure 5Cb). One and two days after CA/ROSC, TNFα immunoreactivity was markedly increased (Figure 5C(c,d)), showing that the ROD of the TNFα immunoreactivity was 205.6% and 248.8% of the Sham-vehicle group, respectively, one day and two days after CA/ROSC (Figure 5D).

TNFα immunoreactivity shown in the tubules of the Sham-Risp group was not different from that shown in the Sham-vehicle group (Figure 5Ce). In the CA-Risp group, TNFα immunoreactivity was also increased in the tubules after CA/CPR (Figure 5C(f–h)), but the immunoreactivities were significantly lower (70.9% and 73.1% of CA-vehicle group one and two days, respectively, after CA/ROSC) than that in the CA-vehicle group (Figure 5D).

### 3.5. Anti-Inflammatory Cytokine Immunoreactivity

#### 3.5.1. IL4 Immunoreactivity

**Figure 6:**
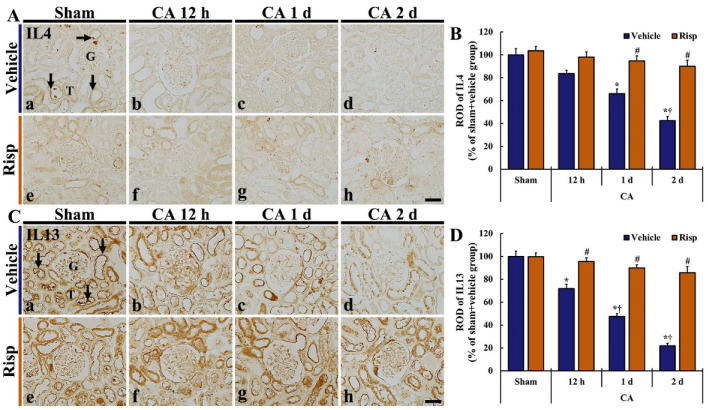
(**A**,**C**) Immunohistochemical staining of IL4 (**A**) and IL13 (**C**) in the kidney obtained from the Sham-vehicle (**a**), CA-vehicle (**b**–**d**), Sham-Risp (**e**) and CA-Risp (**f**–**h**) groups at 12 h, one day and two days after CA/ROSC. In the CA-vehicle group, IL4 and IL13 immunoreactivities (arrows) are gradually decreased after CA/CPR: two days after CA/ROSC, IL4 immunoreactivity is hardly shown and IL13 immunoreactivity is weak. However, IL4 and IL13 immunoreactivities of the CA-Risp group are maintained after CA/ROSC. G, glomerulus; T, tubule. Scale bar = 100 µm. (B and D) RODs of IL4 (**B**) and IL13 (**D**) immunoreactivities. The bars indicate the means ± SEM (*n* = 7, respectively; * *p* < 0.05 vs. Sham-vehicle group; ^#^ *p* < 0.05 vs. CA-vehicle group; ^†^ *p* < 0.05 vs. pre-time point of corresponding group).

Weak IL4 immunoreactivity was mainly observed in the renal tubules of the Sham-vehicle group (Figure 6Aa). In the CA-vehicle group, IL4 immunoreactivity was gradually decreased after CA/CPR, and, on day 2 after CA/ROSC, the immunoreactivity was very low (42.6% of the Sham-vehicle group) (Figure 6A(b–d),B).

In the Sham-Risp group, IL4 immunoreactivity in the tubules was similar to that in the Sham-vehicle group (Figure 6Ae). In the CA-Risp group, IL4 immunoreactivity was slightly decreased after CA/ROSC (Figure 6A(f–h)), showing that the ROD of the IL4 immunoreactivity was 94.7% and 90.0% of the Sham-vehicle group, respectively, on day 1 and 2 after CA/ROSC (Figure 6B).

#### 3.5.2. IL13 Immunoreactivity

Strong IL13 immunoreactivity was easily found in the kidney of the Sham-vehicle group: the IL13 immunoreactivity was generally shown in the tubules (Figure 6Ca). However, IL13 immunoreactivity in the CA-vehicle group was gradually and dramatically reduced after CA/ROSC, showing that showing that the ROD of the IL13 immunoreactivity was 47.5% and 21.8% of the Sham-vehicle group, respectively, one day and two days after CA/ROSC (Figure 6C(b–d),D).

IL13 immunoreactivity in the Sham-Risp group was similar to that obtained from the Sham-vehicle group (Figure 6Ce,D). In the CA-Risp group, IL13 immunoreactivity was not significantly altered after CA/ROSC (Figure 6A(f–h),D).

## 4. Discussion

In the present study, histopathological alteration and changes in the immunoreactivities of pro- and anti-inflammatory cytokines were found in rat kidney due to IRI induced by CA/ROSC. In addition, therapeutic administration of Risp after CA/ROSC significantly attenuated histopathological damage and controlled the increases of pro-inflammatory cytokine immunoreactivities and the decreases of anti-inflammatory cytokine immunoreactivities.

The recovery rate from acute renal injury after out-of-hospital CA is 39%, and recovery from acute renal injury is a strong predictor of survival and good neurological outcomes at discharge from the hospital [37]. Acute renal injury occurs in 30–50% of the patients who survive from CA/ROSC [10] and complicates 12–40% of hospitalized CA patients [38]. Renal injury resulting from CA/ROSC is a complicated process and related with a high mortality rate [39]. Therefore, it is important to investigate acute kidney injury following CA/ROSC. However, many studies have focused on brain and heart injury following CA/ROSC, while renal failure following CA/ROSC has not been largely studied [40]. It is well accepted that, in animal studies, the heart and brain are the most affected organs following IRI induced by CA/ROSC [41,42]. In this study using a rat model of CA/ROSC, the survival rate decreased time-dependently and reached to 6.25% two days after CA/ROSC. It has been reported that, at the early-phase in the post CA syndrome, the survival rate in patients is 4% to 33% [43].

In this study, histopathological damage in the rat kidney with CA/ROSC-induced IRI by histopathological score (tubular injury score and glomeruli lesion score), including the dilatation of the renal tubules, loss of the brush border of the tubules, tubular necrosis, vacuoles in tubular cells, glomerular capillary dilatation, and inflammatory cell infiltration, which were severe two days after CA/ROSC. These findings showed consistency with the results of the previous studies using rats with IRI [44,45]. Taken together, we suggest that the transient block of blood supply to the kidney can evoke severe structural alteration (damage) in the kidney.

Physical hypothermia (i.e., surface cooling) can lead to protection and improve functional impairment or damage in animal models of brain and spinal cord IRI [46,47,48,49,50], although clinical data concerning the effects of hypothermia on brain and spinal cord IRI are disputed. Nevertheless, several studies have reported that hypothermia reduces the severity of renal injury and increases the survival rate in rat models of CA/ROSC [44,51]. In this regard, we need the experiments of the effects of hypothermia-inducing drugs in major organs (i.e., brain, spinal cord, heart, liver, and kidney) of animal models of IRI. It has been demonstrated that Risp exhibits hypothermia [21,23,52]. For several decades, Risp has been widely used for the treatment of schizophrenia [19,20]. Risp, as a second-generation antipsychotic drug, is a benzoxazole derivative and a selective monoaminergic antagonist having high affinity for serotonin type 2 and dopamine type 2 receptors in the limbic system [19,20]. We reported that hypothermia induced by Risp revealed an effective protection of hippocampal neurons from IRI following transient forebrain ischemia in gerbils [23] and positive effects in ischemic liver and spinal cord following CA/ROSC in rats [24,34]. Our present study showed for the first time the beneficial effects of Risp on acute renal IRI following CA/ROSC in rats. Risp induced hypothermia and significantly attenuated histopathological injury in the renal tissues of the rats with CA/ROSC. In addition, the serum levels of renal injury markers (BUN, creatinine, and LDH) were significantly suppressed in the rats with CA/ROSC by Risp administration. Based on our results, we strongly suggest that Risp treatment after renal IRI improves renal damage and dysfunction in patients suffering from CA/ROSC.

Over the past few years, in kidney IRI, pro-inflammatory cytokines enhance kidney damage and contribute to cell death (apoptosis and necrosis) in the tubules [53,54]. In recent years, TNF-α has been well-established as an essential mediator of kidney IRI [55,56]. While renal TNFα expression mediates neutrophil infiltration and injury after renal ischemic insult [57], the mechanisms of TNFα-induced kidney injury are multiple [58,59,60,61]. For example, it has reported that TNFα plays a key role in inflammation after kidney ischemia and reperfusion by up-regulating inflammatory genes [62], and that the inhibition of TNFα activity has antioxidant and anti-inflammatory effects, and protects kidneys from IRI [63]. For the function of IL6 in renal IRI, IL6 emission promotes the expression of oxidative stress and enhances the degree of renal injury including inflammation [64,65]. In acute kidney injury in mice, IL6 can also be upregulated and released from the epithelial cells of the renal tubules in response to the injury and plays an essential role for renal pathophysiology [66]. Additionally, IL6 production is enhanced from the myocardium in states of cardiac ischemia, congestive heart failure, and congenital heart disease in humans and mice [66,67,68]. These findings suggest that pro-inflammatory cytokines can contribute to cell death in the kidney after CA/ROSC. In this regard, we found, in this study, that the immunoreactivities of TNFα and IL6 were significantly increased in the renal tubular cells of the CA-vehicle group as compared with the Sham + vehicle group. However, the immunoreactivities were controlled in the CA-Risp group. Thus, our current findings indicate that Risp can prevent the abnormal increases of TNFα and IL6 in ischemic kidney induced by CA/ROSC.

On the other hand, renal tissue damage can be alleviated by an anti-inflammatory response. Yokota et al. [69] have shown that the deletion of IL4 leads to markedly worse renal function and tubular injury in murine renal IRI. In addition, Jayaraj et al. [70] have reported that endogenous IL13 controls brain inflammation induced by lipopolysaccharide treatment in rats by inhibiting pro-inflammatory cytokine expression, resulting in an enhancement of neuronal survival [70]. These results collectively support the beneficial effects of IL4 and IL13 as anti-inflammatory cytokines. In our current research, significant decreases in immunoreactivities of IL-4 and IL-13 were observed in the kidney of the CA-vehicle group, but, unlike the immunoreactivities of TNFα and IL6, the IL4 and IL13 immunoreactivities in the CA-Risp group were not significantly reduced when compared with the Sham + vehicle group. Our findings are supported by some papers showing that maintained expressions of endogenous anti-inflammatory cytokines, such as IL4 and IL13, contribute to neuronal survival from brain IRI in gerbils [71,72], and that IL4 and IL13 suppress the abnormal expression and production of pro-inflammatory cytokines, such as TNF-α and IL-6, in human monocytes [73,74,75]. Therefore, the maintained expression of anti-inflammatory cytokines (IL4 and IL13) in the kidney of the CA-Risp group may be related with the protective effect of Risp against renal IRI following CA/ROSC.

## 5. Conclusions

This study revealed that CA/ROSC in rats showed high mortality with severe histopathological injury, the increases of renal injury markers (BUN, creatinine, and LDH) and the expressions of TNFα and IL6, and the decrease of IL4 and IL13 expressions in ischemic kidney induced by CA/ROSC. However, Risp administration after CA/ROSC significantly improved the survival rate, reduced the increased expressions of pro-inflammatory cytokines (TNFα and IL6), and maintained the expressions of anti-inflammatory cytokines (IL4 and IL13), ameliorating the renal injury induced by CA/ROSC. These results indicate that Risp treatment after CA/ROSC reduces kidney damage, which is closely associated with the attenuation of renal inflammation following CA/ROSC after CA. Nevertheless, the exact role of Risp is still unclear in the protection of the kidney from IRI following CA/ROSC. Further study is needed to more identify the protective mechanisms of Risp in the kidney after CA/ROSC.

## Figures and Tables

**Table 1:** Primary and secondary antibodies for immunohistochemical staining.

Primary Antibody	Dilution	Function	Supplier
Rabbit anti-interleukin-2 (IL2)	1:500	Proinflammatory cytokine Proinflammatory cytokine	Santa Cruz Biotechnology, Santa Cruz, CA, USA
Rabbit anti-tumor necrosis factor-α (TNFα)	1:1000		Abcam, Cambridge, UK
Rabbit anti-interleukin-4 (IL4)	1:250	Antiinflammatory cytokine Antiinflammatory cytokine	Santa Cruz Biotechnology, Santa Cruz, CA, USA
Rabbit anti-interleukin-13 (IL13)	1:250		Santa Cruz Biotechnology, Santa Cruz, CA, USA
**Secondary antibody**	**Dilution**		**Supplier**
Biotinylated goat anti-rabbit IgG	1:300		Vector Laboratories Inc., Burlingame, CA, USA

## Data Availability

The data presented in this study are available on request from the corresponding authors.
